# Physical Activity, Outcome Expectations, and Social Support in Dyads of Frail Older Adults and Their Home Care Aides

**DOI:** 10.1093/geroni/igaa057.1299

**Published:** 2020-12-16

**Authors:** Lijuan Yin, Naoko Muramatsu

**Affiliations:** University of Illinois at Chicago, Chicago, Illinois, United States

## Abstract

Frail community-dwelling older adults increasingly receive home care and continue to face barriers to participating in physical activity (PA) that could help maintain their function. Home care aides (HCAs) are well-positioned to promoting PA among older home care recipients because of their established relationship and regular interpersonal exchanges; yet, the role of HCAs in promoting and supporting PA in home care settings is seldomly studied. Using the quantitative and qualitative data from a 4-month home-based gentle PA intervention delivered by HCAs to their clients in a Medicaid-funded home care setting, the current study examined whether outcome expectations for exercise (OEE) held by HCAs led to client PA outcomes (i.e. functional limitations and physical performance) through social support for exercise (SSE) provided by HCAs. Longitudinal mediation analysis of 46 HCA-client dyads showed that higher baseline OEE held by HCAs were related to greater SSE reported by clients after the intervention (p<.05; bootstrapped standard errors), controlling for client-level covariates, including baseline OEE, age, gender, comorbidity, and whether HCA was client’s family member. Unexpectedly, SSE did not have significant association with client PA outcomes nor mediated the relationship between OEE held by HCAs and client PA outcomes. Qualitative data suggested alternative factors may explain the results, such as clients’ family beliefs in the intervention and clients’ participation experiences (such as expectation fulfillment). Future research should consider older home care clients’ family contexts to enhance our understanding of HCAs’ roles in preserving the function of growing numbers of older home care recipients.

